# Reduced Data Sets and Entropy-Based Discretization

**DOI:** 10.3390/e21111051

**Published:** 2019-10-28

**Authors:** Jerzy W. Grzymala-Busse, Zdzislaw S. Hippe, Teresa Mroczek

**Affiliations:** 1 Department of Electrical Engineering and Computer Science, University of Kansas, Lawrence, KS 66045, USA; 2 Department of Artificial Intelligence, University of Information Technology and Management, 35–225 Rzeszow, Poland; zhippe@wsiz.rzeszow.pl (Z.S.H.); tmroczek@wsiz.rzeszow.pl (T.M.)

**Keywords:** data mining, numerical attributes, discretization, entropy

## Abstract

Results of experiments on numerical data sets discretized using two methods—global versions of Equal Frequency per Interval and Equal Interval Width-are presented. Globalization of both methods is based on entropy. For discretized data sets left and right reducts were computed. For each discretized data set and two data sets, based, respectively, on left and right reducts, we applied ten-fold cross validation using the C4.5 decision tree generation system. Our main objective was to compare the quality of all three types of data sets in terms of an error rate. Additionally, we compared complexity of generated decision trees. We show that reduction of data sets may only increase the error rate and that the decision trees generated from reduced decision sets are not simpler than the decision trees generated from non-reduced data sets.

## 1. Introduction

The problem of reducing (or selecting) the set of attributes (or features) is known for many decades [1,2,3,4,5,6]. It is a central topic in multivariate statistics and data analysis [7]. It was recognized as a variant of the Set Covering problem in [3]. The problem of finding a minimal set of features is crucial for the study of medical and bioinformatics data, with tens of thousands of features representing genes [7]. An analysis of feature selection methods, presented in [7], included filter, wrapper and embedded methods, based on mathematical optimization, e.g., on linear programming. An algorithm for discretization that removes redundant attributes was presented in [8,9,10]. An example of a MicroArray Logic Analyzer (MALA), where feature selection was based on cluster analysis, was presented in [11]. An approach to feature selection, based on logic, was presented in [12]. A lot of attention has been paid to benefits of feature reduction in data mining, machine learning and pattern recognition, see for example, References [4,5,6,13,14,15,16,17]. Recently, feature reduction or reducing of the attribute set, combined with discretization of numerical attributes, was discussed in Reference [13,15,16,18,19]. In Reference [18], results of experiments conducted on ten numerical data sets with four different types of reducts were presented. The authors used two classifiers, Support Vector Machine (SVM) [20] and C4.5 [21]. In both cases, results of the Friedman test are inconclusive, so benefits of data reduction are not clear. In experiments presented in Reference [19], genetic algorithms and artificial neural networks were used for a few tasks: discretization, feature reduction and prediction of stock price index. It is difficult to evaluate how feature reduction contributed to final results. In Reference [13] experimental results on ten numerical data sets, discretized using a system called C-GAME, are reported. C-GAME uses reducts during discretization. The authors claim that C-GAME outperforms five other discretization schemes. Some papers, for example, Reference [14,15,16], discuss reducts combined with discretization. However, no convincing experimental results are included. A related problem, namely how reduction of the attribute set as a side-effect of discretization of numerical attributes changes an error rate, was discussed in Reference [17].

For symbolic attributes, it was shown [22] that the quality of rule sets induced from reduced data sets, measured by an error rate evaluated by ten-fold cross validation, is worse than the quality of rule sets induced form the original data sets, with no reduction of the attribute set.

## 2. Reducts

The set of all cases of the data set is denoted by *U*. An example of the data set with numerical attributes is presented in Table 1. For simplicity, all attributes have repetitive values, though in real-life numerical attribute values are seldom repetitive. In our example *U* = {1, 2, 3, 4, 5, 6, 7, 8}. The set of all attributes is denoted by *A*. In our example *A* = {*Length*, *Height*, *Width*, *Weight*}. One of the variables is called a *decision*, in Table 1 it is *Quality*.

Let *B* be a subset of the set *A* of all attributes. The *indiscernibility relation* IND(B) [23,24] is defined as follows

(x,y)∈IND(B) if and only if a(x)=a(y) for anya∈B,

where x,y∈U and a(x) denotes the value of an attribute a∈A for a case x∈U. The relation IND(B) is an equivalence relation. An equivalence class of IND(B), containing x∈U, is called a *B*-*elementary class* and is denoted by ${\left[x\right]}_{B}$. A family of all sets ${\left[x\right]}_{B}$, where x∈U, is a partition on *U* denoted by $B^{*}$. A union of *B*-elementary classes is called *B*-*definable*. For a decision *d* we may define an indiscernibility relation IND{d} by analogy. Additionally, {*d*}-elementary classes are called *concepts*.

A decision *d* *depends* on the subset *B* of the set *A* of all attributes if and only if $B^{*}\leq {\left\{d\right\}}^{*}$. For partitions π and τ on *U*, π≤τ if and only if for every Y∈τ there exists X∈π such that X⊆Y. For example, for *B* = {*Width*, *Weight*}, $B^{*}$ = {{1}, {2, 3}, {4}, {5}, {6, 7, 8}}, ${\left\{d\right\}}^{*}$ = {{1, 2, 3}, {4}, {5, 6, 7, 8}} and $B^{*}\leq {\left\{d\right\}}^{*}$. Thus *d* depends on *B*.

For Table 1 and for *B* = {*Weight*}, $B^{*}$ = {{1, 2, 3}, {4}, {5, 6, 7, 8}}. The concepts {4, 5}, {6, 7, 8} are not *B*-definable. For an undefinable set *X* we define two definable sets, called *lower* and *upper* approximations of *X* [23,24]. The lower approximation of *X* is defined as follows

$$\left\{x\;|\;x\in U,{\left[x\right]}_{B}\subseteq X\right\}$$

and is denoted by $\underline{B}X$. The upper approximation of *X* is defined as follows

$$\left\{x\;|\;x\in U,{\left[x\right]}_{B}\cap X\neq \emptyset \right\}$$

and is denoted by $\bar{B}X$. For Table 1 and *B* = {Weight}, $\underline{B}${6, 7, 8} = ∅ and $\bar{B}${6, 7, 8} = {5, 6, 7, 8}.

A set *B* is called a *reduct* if and only if *B* is the smallest set with $B^{*}\leq {\left\{d\right\}}^{*}$. The set {*Width*, *Weight*} is the reduct since {*Width*}${}^{*}$ = {{1, 4, 5}, {2, 3, 6, 7, 8}} $≰{\left\{d\right\}}^{*}$ and {*Weight*}${}^{*}$ = {{1, 2, 3}, {4}, {5, 6, 7, 8}} $≰{\left\{d\right\}}^{*}$, so *B* is the smallest set with $B^{*}\leq {\left\{d\right\}}^{*}$.

An idea of the reduct is important since we may restrict our attention to a subset *B* and construct a decision tree with the same ability to distinguish all concepts that are distinguishable in the data set with the entire set *A* of attributes. Note that any algorithm for finding all reducts is of exponential time complexity. In practical applications, we have to use some heuristic approach. In this paper, we suggest two such heuristic approaches, left and right reducts.

A *left reduct* is defined by a process of a sequence of attempts to remove one attribute at a time, from right to left and by checking after every attempt whether $B^{*}\leq {\left\{d\right\}}^{*}$, where *B* is the current set of attributes. If this condition is true, we remove an attribute. If not, we put it back. For the example presented in Table 1, we start from an attempt to remove the rightmost attribute, that is, *Weight*. The current set *B* is {*Length*, *Height*, *Width*}, $B^{*}$ = {{1}, {2, 3}, {4}, {5}, {6}, {7, 8}} $\leq {\left\{d\right\}}^{*}$, so we remove *Weight* for good. The next candidate for removal is *Width*, the set *B* = {*Length*, *Height*}, $B^{*}$ = {{1}, {2, 3}, {4}, {5}, {6}, {7, 8}} and $B^{*}\leq {\left\{d\right\}}^{*}$, so we remove *Width* as well. The next candidate is *Height*, if we remove it, *B* = {*Length*} $\leq {\left\{d\right\}}^{*}$, so {*Length*} is the left reduct since it cannot be further reduced.

Similarly, a *right reduct* is defined by a similar process of a sequence of attempts to remove one attribute at a time, this time from left to right. Again, after every attempt we check whether $B^{*}\leq {\left\{d\right\}}^{*}$. It is not difficult to see that the right reduct is the set {*Width*, *Weight*}.

For a discretized data set we may compute left and right reducts, create three data sets: with the discretized (non-reduced) data set and with attribute sets restricted to the left and right reducts and then for all three data sets compute an error rate evaluated by C4.5 decision tree generation system using ten-fold cross validation. Our results show again that reduction of data sets causes increase of an error rate.

## 3. Discretization

For a numerical attribute *a*, let $a_{i}$ be the smallest value of *a* and let $a_{j}$ be the largest value of *a*. In discretizing of *a* we are looking for the numbers $a_{i_{0}}$, $a_{i_{1}}$, ..., $a_{i_{k}}$, called *cutpoints*, where $a_{i_{0}}=a_{i}$, $a_{i_{k}}=a_{j}$, $a_{i_{l}}$ < $a_{i_{l+1}}$ for *l* = 0, 1,..., k-1 and *k* is a positive integer. As a result of discretization, the domain $\left[a_{i},a_{j}\right]$ of the attribute *a* is divided into *k* intervals

$$\{\left[a_{i_{0}},a_{i_{1}}\right),\left[a_{i_{1}},a_{i_{2}}\right),...,\left[a_{i_{k-2}},a_{i_{k-1}}\right),\left[a_{i_{k-1}},a_{i_{k}}\right]\}.$$

In this paper we denote such intervals as follows

$$a_{i_{0}}..a_{i_{1}},a_{i_{1}}..a_{i_{2}},...,a_{i_{k-2}}..a_{i_{k-1}},a_{i_{k-1}}..a_{i_{k}}.$$

Discretization is usually conducted not on a single numerical attribute but on many numerical attributes. Discretization methods may be categorized as supervised or decision-driven (concepts are taken into account) or unsupervised. Discretization methods processing all attributes are called global or dynamic, discretization methods processing a single attribute are called local or static.

Let *v* be a variable and let $v_{1}$, $v_{2}$, ..., $v_{n}$ be values of *v*, where *n* is a positive integer. Let *S* be a subset of *U*. Let $p(v_{i})$ be a probability of $v_{i}$ in *S*, where *i* = 1, 2, ..., n. An *entropy* $H_{S}\left(v\right)$ is defined as follows

$$H_{S}\left(v\right)=-\sum_{i=1}^{n}p\left(v_{i}\right)\cdot \log p\left(v_{i}\right).$$

All logarithms in this paper are binary.

Let *a* be an attribute, let $a_{1}$, $a_{2}$, ..., $a_{m}$ be all values of *a* restricted to *S*, let *d* be a decision and let $d_{1}$, $d_{2}$, ..., $d_{n}$ be all values of *d* restricted to *S*, where *m* and *n* are positive integers. A conditional entropy $H_{S}\left(d|a\right)$ of the decision *d* given an attribute *a* is defined as follows

$$-\sum_{j=1}^{m}p\left(a_{j}\right)\cdot \sum_{i=1}^{n}p\left(d_{i}|a_{j}\right)\cdot log\;p\left(d_{i}|a_{j}\right),$$

where $p(d_{i}|a_{j})$ is the conditional probability of the value $d_{j}$ of the decision *d* given $a_{j}$; j∈{1,2,...,m} and i∈{1,2,...,n}.

As is well-known [25,26,27,28,29,30,31,32,33,34,35,36], discretization that uses conditional entropy of the decision given attribute is believed to be one of the most successful discretization techniques.

Let *S* be a subset of *U*, let *a* be an attribute and let *q* be a cutpoint splitting the set *S* into two subsets $S_{1}$ and $S_{2}$. The corresponding conditional entropy, denoted by $H_{S}\left(d|a\right)$ is defined as follows

$$\frac{|S_{1}|}{\left|U\right|}H_{S_{{}_{1}}}\left(a\right)+\frac{|S_{2}|}{\left|U\right|}H_{S_{2}}\left(a\right),$$

where |X| denotes the cardinality of the set *X*. Usually, the cutpoint *q* for which $H_{S}\left(d|a\right)$ is the smallest is considered to be the best cutpoint.

We need how to halt discretization. Commonly, we halt discretization when we may distinguish the same cases in the discretized data set as in in the original data set with numerical attributes. In this paper discretization is halted when the *level of consistency* [26], defined as follows

$$L\left(A\right)=\frac{\sum_{X\in {\left\{d\right\}}^{*}}\left|\underline{A}X\right|}{\left|U\right|}$$

and denoted by L(A), is equal to 1. For Table 1, $A^{*}$ = {{1}, {2, 3}, {4}, {5}, {6}, {7, 8}}, so $\underline{A}X$ = *X* for any concept *X* from ${\left\{d\right\}}^{*}$. On the other hand, for *B* = {*Weight*},

$$L\left(B\right)=\frac{|\underline{B}\left\{1,2,3\right\}|+|\underline{B}\left\{4,5\right\}|+|\underline{B}\left\{6,7,8\right\}|}{\left|U\right|}=\frac{\left|\{1,2,3\}|+|\emptyset |+|\emptyset \right|}{8}=0.375.$$

## 4. Equal Frequency per Interval and Equal Interval Width

Both discretization methods, Equal Frequency per Interval and Equal Interval Width, are frequently used in discretization and both are known to be efficient [25]. In local versions of these methods, only a single numerical attribute is discretized at a time [31]. The user provides a parameter denoted by *k*. This parameter is equal to a requested number of intervals. In the Equal Frequency per Interval method, the domain of a numerical attribute is divided into *k* intervals with approximately equal number of cases. In the Equal Interval Width method, the domain of a numerical attribute is divided into *k* intervals with approximately equal width.

In this paper we present a supervised and global version of both methods, based on entropy [26]. Using this idea, we start from discretizing all numerical attributes assuming *k* = 2. Then the level of consistency is computed for the data set with discretized attributes. If the level of consistency is sufficient, discretization ends. If not, we select the worst attribute for additional discretization. The measure of quality of the discretized attribute, denoted by $a^{d}$ and called the *average block entropy*, is defined as follows

$$M\left(a^{d}\right)=\frac{\sum_{B\in {\left\{a^{d}\right\}}^{*}}\frac{\left|B\right|}{\left|U\right|}H\left(B\right)}{|{\left\{a^{d}\right\}}^{*}|}.$$

A discretized attribute with the largest value of $M(a^{d})$ is the worst attribute. This attribute is further discretized into k+1 intervals. The process is continued by recursion. The time computational complexity, in the worst case, is $O(m\cdot logm\cdot n^{2})$, where *m* is the number of cases and *n* is the number of attributes. This method is illustrated by applying the Equal Frequency per Interval method for the data set from Table 1. Table 2 presents the discretized data set for the data set from Table 1. It is not difficult to see that the level of consistency for Table 2 is 1.

For the data set presented in Table 2, both left and right reducts are equal to each other and equal to {Height${}^{d}$, Width${}^{d}$, Weight${}^{d}$}.

Table 3 presents the data set from Table 1 discretized by the Global Equal Interval Width discretization method. Again, the level of consistency for Table 3 is equal to 1. Additionally, for the data set from Table 3, both reducts, left and right, are also equal to each other and equal to {Length${}^{d}$, Width${}^{d}$}.

## 5. Experiments

We conducted experiments on 13 numerical data sets, presented in Table 4. All of these data sets may be accessed in *Machine Learning Repository*, University of California, Irvine, except for *bankruptcy*. The *bankruptcy* data set was described in Reference [37].

The main objective of our research is to compare the quality of decision trees generated by C4.5 directly from discretized data sets and from data sets based on reducts, in terms of an error rate and tree complexity. Data sets were discretized by the Global Equal Frequency per Interval and Global Equal Interval Width methods with the level of complexity equal to 1. For each numerical data set three data sets were considered:

- an original (non-reduced) discretized data set,
- a data set based on the left reduct of the original discretized data set and
- a data set based on right reduct of the original discretized data set.

The discretized data sets were inputted to the C4.5 decision tree generating system [21]. In our experiments, the error rate was computed using an internal mechanism of the ten-fold cross validation of C4.5.

Additionally, an internal discretization mechanism of C4.5 was excluded in experiments for left and right reducts since in this case data sets were discretized by the global discretization methods, so C4.5 considered all attributes as symbolic.

**Figure 1:**
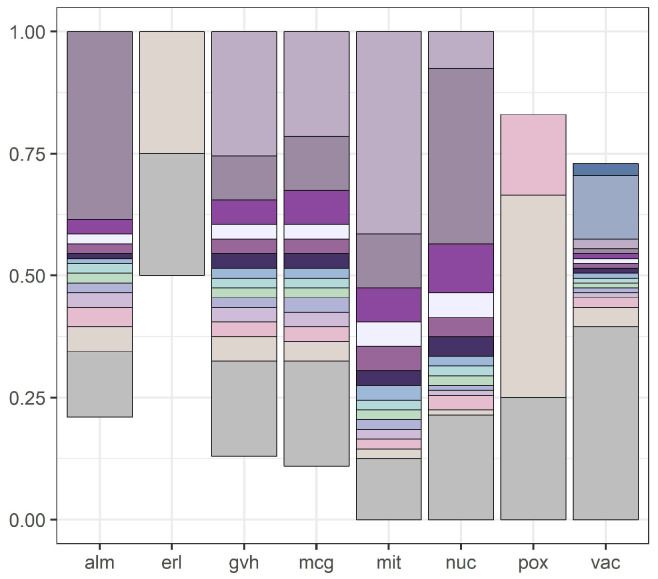
Attribute intervals for the *yeast* data set discretized by the internal discretization mechanism of C4.5.

**Figure 2:**
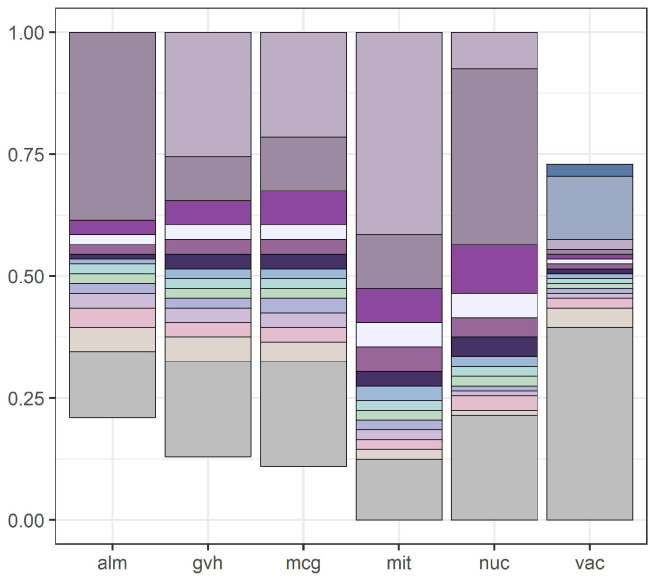
Attribute intervals for the *yeast* data set discretized by the global version of Equal Frequency per Interval method and then computing right reducts.

We illustrate our results with Figure 1 and Figure 2. Figure 1 presents discretization intervals for *yeast* data set, where discretization was conducted by the internal discretization mechanism of C4.5. Figure 2 presents discretization intervals for the same data set with discretization conducted by the global version of the Equal Frequency per Interval method (right reducts and left reducts were identical).

Results of our experiments are presented in Table 5, Table 6, Table 7 and Table 8. These results were analyzed by the Friedman rank sum test with multiple comparisons, with 5% level of significance. For data sets discretized by the Global Equal Frequency per Interval method, the Friedman test shows that there are significant differences between the three types of data sets: the non-reduced discretized data sets and data sets based on left and right reducts. In most cases, the original, non-reduced data sets are associated with the smallest error rates than both left and right reducts. However, the test of multiple comparisons shows that the differences are not statistically significant.

For data sets discretized by the Global Equal Interval Width method results are more conclusive. There are statistically significant differences between non-reduced discretized data sets and data sets based on left and right reducts. Moreover, an error rate for the non-reduced discretized data sets is significantly smaller than for both types of data sets, based on left and right reducts. As expected, the difference between left and right reducts is not significant.

For both discretization methods and all types of data sets (non-reduced, based on left and right reducts) the difference in complexity of generated decision trees, measured by tree size and depth, is not significant.

Table 8 shows the size of left and right reducts created from data sets discretized by the Global versions of Equal Frequency per Interval and Equal Interval Width methods. For some data sets, for example, for *bupa*, both left and right reducts are identical with the original attribute set.

## 6. Conclusions

Our preliminary results [22] show that data reduction combined with rule induction causes an increase of the error rate. The current results, presented in this paper, confirm these results: the reduction of data sets, associated with C4.5 tree generation system, causes the same effect. Decision trees generated from reduced data sets increase the error rate as evaluated by ten-fold cross validation. Additionally, decision trees generated from reduced data sets, in terms of a tree size or tree depth, are not simpler than decision trees generated from non-reduced data sets.Therefore, it is obvious that reduction of data sets (or feature selection) should be used with caution since it may degrade results of data mining.

In the future we are planning to extend our experiments to large data sets and to include other classifiers than systems for rule induction and decision tree generation.

## Figures and Tables

**Table 1:** An example of a data set with numerical attributes.

Case	Attributes				Decision
	Length	Height	Width	Weight	Quality
1	4.8	1.2	1.6	0.8	high
2	4.8	1.4	1.8	0.8	high
3	4.8	1.4	1.8	0.8	high
4	4.4	1.4	1.6	1.0	medium
5	4.4	1.2	1.6	1.4	medium
6	4.2	1.2	1.8	1.4	low
7	4.2	1.8	1.8	1.4	low
8	4.2	1.8	1.8	1.4	low

**Table 2:** A data set discretized by Equal Frequency per Interval.

Case	Attributes				Decision
	Length${}^{d}$	Height${}^{d}$	Width${}^{d}$	Weight${}^{d}$	Quality
1	4.3..4.8	1.2..1.3	1.6..1.7	0.8..1.2	high
2	4.3..4.8	1.3..1.8	1.7..1.8	0.8..1.2	high
3	4.3..4.8	1.3..1.8	1.7..1.8	0.8..1.2	high
4	4.3..4.8	1.3..1.8	1.6..1.7	0.8..1.2	medium
5	4.3..4.8	1.2..1.3	1.6..1.7	1.2..1.4	medium
6	4.2..4.3	1.2..1.3	1.7..1.8	1.2..1.4	low
7	4.2..4.3	1.3..1.8	1.7..1.8	1.2..1.4	low
8	4.2..4.3	1.3..1.8	1.7..1.8	1.2..1.4	low

**Table 3:** A data set discretized by Equal Interval Width.

Case	Attributes				Decision
	Length${}^{d}$	Height${}^{d}$	Width${}^{d}$	Weight${}^{d}$	Quality
1	4.5..4.8	1.2..1.5	1.6..1.7	0.8..1.2	high
2	4.5..4.8	1.2..1.5	1.7..1.8	0.8..1.2	high
3	4.5..4.8	1.2..1.5	1.7..1.8	0.8..1.2	high
4	4.2..4.5	1.2..1.5	1.7..1.8	0.8..1.2	medium
5	4.2..4.5	1.2..1.5	1.7..1.8	1.2..1.4	medium
6	4.2..4.5	1.5..1.8	1.7..1.8	1.2..1.4	low
7	4.2..4.5	1.5..1.8	1.7..1.8	1.2..1.4	low
8	4.2..4.5	1.5..1.8	1.7..1.8	1.2..1.4	low

**Table 4:** Data sets.

Data Set		Number of	
	Cases	Attributes	Concepts
Abalone	4177	8	29
Australian	690	14	2
Bankruptcy	66	5	2
Bupa	345	6	2
Echocardiogram	74	7	2
Ecoli	336	8	8
Glass	214	9	6
Ionosphere	351	34	2
Iris	150	4	3
Leukemia	415	175	2
Wave	512	21	3
Wine Recognition	178	13	3
Yeast	1484	8	9

**Table 5:** Error rates for discretized data sets.

Data Set	Equal Frequency per Interval			Equal Interval Width		
	No Reduction	Left Reducts	Right Reducts	No Reduction	Left Reducts	Right Reducts
Abalone	76.87	76.99	77.47	76.90	77.42	77.42
Australian	12.46	14.49	22.46	13.33	30.14	14.64
Bankruptcy	3.03	3.03	3.03	10.61	10.61	10.61
Bupa	35.94	35.94	35.94	34.49	34.49	34.49
Echocardiogram	27.03	27.03	27.03	31.08	39.19	39.19
Ecoli	30.65	28.87	28.87	28.57	28.57	28.57
Glass	41.12	39.25	41.59	33.18	33.64	33.64
Ionosphere	13.11	19.15	20.85	10.83	11.97	15.82
Iris	12.67	12.67	12.67	4.00	4.00	4.00
Leukemia	1.45	2.93	1.60	1.32	1.99	1.59
Wave	25.59	27.15	28.91	27.54	31.46	28.13
Wine Recognition	10.11	10.67	12.36	9.55	8.99	10.67
Yeast	57.82	57.82	57.82	56.54	56.87	56.87

**Table 6:** Tree size for discretized data sets.

Data Set	Tree Size					
	Equal Frequency per Interval			Equal Interval Width		
	No Reduction	Left Reducts	Right Reducts	No Reduction	Left Reducts	Right Reducts
Abalone	28,236	27,202	24,905	18,711	16,491	16,491
Australian	41	3	12	39	95	3
Bankruptcy	3	3	3	6	6	6
Bupa	17	17	17	27	27	27
Echocardiogram	8	13	13	16	16	16
Ecoli	61	40	40	109	107	107
Glass	70	63	56	186	191	191
Ionosphere	33	53	71	34	24	70
Iris	11	11	11	4	4	4
Leukemia	139	125	132	229	139	174
Wave	62	42	44	107	73	96
Wine Recognition	19	30	30	18	15	18
Yeast	662	678	678	913	914	914

**Table 7:** Tree depth for discretized data sets.

Data Set	Tree Depth					
	Equal Frequency per Interval			Equal Interval Width		
	No Reduction	Left Reducts	Right Reducts	No Reduction	Left Reducts	Right Reducts
Abalone	2	2	2	3	3	3
Australian	5	1	2	5	4	1
Bankruptcy	1	1	1	1	1	1
Bupa	2	2	2	2	2	2
Echocardiogram	2	2	2	3	3	3
Ecoli	3	2	2	3	3	3
Glass	5	4	5	5	5	5
Ionosphere	6	6	5	6	4	6
Iris	2	2	2	1	1	1
Leukemia	6	4	4	6	4	5
Wave	9	7	9	11	9	6
Wine Recognition	6	6	6	5	4	5
Yeast	4	4	4	3	3	3

**Table 8:** Number of attributes in left and right reducts for discretized data sets.

Data Set	Discretized Non-Reduced	Equal Frequency per Interval		Equal Interval Width	
	Data Set	Left Reducts	Right Reducts	Left Reducts	Right Reducts
Abalone	8	6	6	6	6
Australian	14	8	10	9	9
Bankruptcy	5	4	4	4	4
Bupa	6	6	6	6	6
Echocardiogram	7	5	5	6	6
Ecoli	7	5	5	5	5
Glass	9	8	8	7	7
Ionosphere	34	11	13	11	10
Iris	4	4	4	4	4
Leukemia	15	8	8	11	10
Wave	21	15	14	14	14
Wine Recognition	13	8	8	10	10
Yeast	8	6	6	6	6

## References

[1] Almuallim H., Dietterich T.G. (1994). Learning Boolean concepts in the presence of many irrelevant features. Artif. Intell..

[2] Kira K., Rendell L.A. The feature selection problem: Traditional methods and a new algorithm. Proceedings of the 10-th National Conference on AI.

[3] Garey M., Johnson D. (1979). Computers and Intractability: A Guide to the Theory of NP-Completeness.

[4] Fuernkranz J., Gamberger D., Lavrac N. (2012). Foundations of Rule Learning.

[5] Stanczyk U., Jain L.C. (2015). Feature Selection for Data and Pattern Recognition.

[6] Stanczyk U., Zielosko B., Jain L.C. (2018). Advances in Feature Selection for Data and Pattern Recognition.

[7] Bertolazzi P., Felici G., Festa P., Fiscon G., Weitschek E. (2016). Integer programming models for feature selection: New extensions and a randomized solution algorithm. Inf. Sci..

[8] Santoni D., Weitschek E., Felici G. (2016). Optimal discretization and selection of features by association rates of joint distributions. RAIRO-Oper. Res..

[9] Liu H., Rudy S. (1997). Feature selection via discretization. IEEE Trans. Knowl. Data Eng..

[10] Sharmin S., Ali A.A., Khan M.A.H., Shoyaib M. Feature selection and discretization based on mutual information. Proceedings of the IEEE International Conference on Imaging, Vision & Pattern Recognition.

[11] Weitschek E., Felici G., Bertolazzi P. MALA: A microarray clustering and classification software. Proceedings of the International Workshop on Database and Expert Systems Applications.

[12] Felici G., Weitschek E. Mining logic models in the presence of noisy data. Proceedings of the International Symposium on Articial Intelligence and Mathematics.

[13] Tian D., Zeng X.J., Keane J. (2011). Core-generating approximated minimum entropy discretization for rough set feature selection in pattern classification. Int. J. Approx. Reason..

[14] Jensen R., Shen Q. Fuzzy-rough sets for descriptive dimensionality reduction. Proceedings of the International Conference on Fuzzy Systems FUZZ-IEEE 2002.

[15] Nguyen H.S. (1998). Discretization problem for rough sets methods. Proceedings of the 1-st International Conference RSCTC 1998 on Rough Sets and Current Trends in Computing.

[16] Swiniarski R.W. (2001). Rough set methods in feature reduction and classification. Int. J. Appl. Math. Comput. Sci..

[17] Grzymala-Busse J.W., Mroczek T., Stanczyk B., Zielosko B., Jain L.C. (2017). Attribute selection based on reduction of numerical attribute during discretization. Advances in Feature Selection for Data and Pattern Recognition.

[18] Hu Q., Yu D., Xie Z. (2006). Information-preserving hybrid data reduction based on fuzzy-rough techniques. Pattern Recognit. Lett..

[19] Kim K.j., Han I. (2000). Genetic algorithms approach to feature discretization in artificial neural networks for the prediction of stock price index. Expert Syst. Appl..

[20] Cortes C., Vapnik V. (1995). Support-vector networks. Mach. Learn..

[21] Quinlan J.R. (1993). C4.5: Programs for Machine Learning.

[22] Grzymala-Busse J.W., Greco S., Bouchon-Meunier B., Coletti G., Fedrizzi M., Matarazzo B., Yager R.R. (2012). An empirical comparison of rule induction using feature selection with the LEM2 algorithm. Communications in Computer and Information Science.

[23] Pawlak Z. (1982). Rough sets. Int. J. Comput. Inf. Sci..

[24] Pawlak Z. (1991). Rough Sets. Theoretical Aspects of Reasoning about Data.

[25] Blajdo P., Grzymala-Busse J.W., Hippe Z.S., Knap M., Mroczek T., Piatek L. A comparison of six approaches to discretization—A rough set perspective. Proceedings of the Rough Sets and Knowledge Technology Conference.

[26] Chmielewski M.R., Grzymala-Busse J.W. (1996). Global discretization of continuous attributes as preprocessing for machine learning. Int. J. Approx. Reason..

[27] Clarke E.J., Barton B.A. (2000). Entropy and MDL discretization of continuous variables for Bayesian belief networks. Int. J. Intell. Syst..

[28] Elomaa T., Rousu J. (1999). General and efficient multisplitting of numerical attributes. Mach. Learn..

[29] Fayyad U.M., Irani K.B. (1992). On the handling of continuous-valued attributes in decision tree generation. Mach. Learn..

[30] Fayyad U.M., Irani K.B. Multi-interval discretization of continuous-valued attributes for classification learning. Proceedings of the Thirteenth International Joint Conference on Artificial Intelligence.

[31] Grzymala-Busse J.W., Kloesgen W., Zytkow J. (2002). Discretization of numerical attributes. Handbook of Data Mining and Knowledge Discovery.

[32] Grzymala-Busse J.W. A multiple scanning strategy for entropy based discretization. Proceedings of the 18th International Symposium on Methodologies for Intelligent Systems.

[33] Kohavi R., Sahami M. Error-based and entropy-based discretization of continuous features. Proceedings of the Second International Conference on Knowledge Discovery and Data Mining.

[34] Nguyen H.S., Nguyen S.H., Polkowski L., Skowron A. (1998). Discretization methods in data mining. Rough Sets in Knowledge Discovery 1: Methodology and Applications.

[35] Stefanowski J. Handling continuous attributes in discovery of strong decision rules. Proceedings of the First Conference on Rough Sets and Current Trends in Computing.

[36] Stefanowski J. (2001). Algorithms of Decision Rule Induction in Data Mining.

[37] Altman E.I. (1968). Financial ratios, discriminant analysis and the prediction of corporate bankruptcy. J. Financ..

